# Monthly and seasonal prevalence of asthma and chronic obstructive pulmonary disease in the District Dera Ismail Khan, Khyber Pakhtunkhwa, Pakistan

**DOI:** 10.1186/s43168-022-00166-2

**Published:** 2022-12-03

**Authors:** Muhammad Ashraf Khan

**Affiliations:** Elementary and Secondary Education Department, Khyber Pakhtunkhwa, Pakistan

**Keywords:** Asthma, Chronic obstructive pulmonary disease, Cases, Winter

## Abstract

**Background:**

Asthma and chronic obstructive pulmonary disease are the major airway diseases and are increasingly important causes of mortality, morbidity, and disability globally. This cross-sectional study was conducted to determine monthly and seasonal patterns of asthma and chronic obstructive pulmonary disease in the study area during 2020–2021. The data of the indoor patients for the study period were obtained from the official records maintained in the District Head Quarter teaching hospital Dera Ismail Khan.

**Results:**

A total of 2179 cases of asthma were recorded including 1609 (73.8%) in 2020 and 570 (26.2%) in 2021, and 740 cases of the chronic obstructive pulmonary disease were also recorded in 2020. Overall asthma was highest in March with 1128 (51.8%) cases and was lowest in July with 29 (1.3%) cases. Seasonal prevalence/pattern show that asthma relatively more prevailed during winter and early spring (December through March). Out of a total of 740 cases of chronic obstructive pulmonary disease, the highest 405 (54.7%) cases were recorded in February and the lowest 0 in April. Seasonal distribution indicated that chronic obstructive pulmonary disease was relatively higher in winter (January–February).

**Conclusions:**

Seasonal variation and pattern in prevalence exist both in asthma and chronic obstructive pulmonary disease as both indicated relatively higher prevalence in winter compared to summer and autumn.

## Background

Five major respiratory diseases are asthma, chronic obstructive pulmonary disease (COPD), lung cancer, pneumonia, and tuberculosis, and they have contributed to 20% of global mortality [1]. Both asthma and COPD are the most common and most important chronic respiratory diseases globally [2–4].

Asthma is a chronic inflammatory disorder of the airways characterized by episodes of reversible breathing problems due to narrowing and obstruction of the bronchus and bronchioles [5]. Further asthma is characterized by recurrent episodes of breathlessness, wheezing, chest tightness, cough, and sputum production [6–9]. It is typically caused by a long-term exposure to irritating gases or particulate matter, most often from cigarette smoke [10].

COPD is progressive airflow limitation caused by chronic inflammation of the bronchus/bronchioles and lung parenchyma [8] and is symptomized with shortness of breath, wheezing, chest tightness, and chronic cough and sputum production [8, 11], The leading risk factors for COPD are tobacco smoking and exposure to indoor air pollution, ambient air pollution, and occupational pollutants [12, 13].

Three hundred thirty-nine million people are suffering from asthma and around 1000 people die per day of asthma globally [7, 14], while two hundred fifty-one million people are suffering from COPD globally [11]. COPD is a major cause of death worldwide [12, 15] and led to around 90% mortality in low- or middle-income countries [16] and by 2030 COPD, and related conditions will be expected to result in 4.5 million deaths globally [17]. Fifteen million children and 7.5 million adults are suffering from asthma in Pakistan [10, 18]. Asthma and COPD are estimated to occur in Pakistan are 4.3% and 2.1%, respectively [19]. In Pakistan, both asthma and COPD are major respiratory problems and resulted in one fourth of patients at primary healthcare (PHC) facilities [20].

Seasonal dynamics in the prevalence of diseases have epidemiological significance [21]. The present study is the first on the monthly/seasonal prevalence of asthma in Khyber Pakhtunkhwa (KP) and the first on COPD in Pakistan.

## Methods

### Study design, data collection, and setting

This cross-sectional study reported the data of indoor patients in the District Head Quarter (DHQ) teaching hospital Dera Ismail Khan (D.I.Khan). The data were obtained from hospital computerized records from the administration (computer section) of the said hospital. Data on both asthma and COPD were converted into monthly percentage prevalence, and relative prevalence was determined.

### Diagnosis of asthma and COPD

The patients with symptoms of asthma and COPD are diagnosed in the DHQ hospital D.I.Khan and are admitted to the hospital. Both chest radiography (chest X-ray) and chest computed tomography (CT) are the primary imaging methods to diagnose asthmatic and COPD patients in the district. Both CT and magnetic resonance imaging (MRI) are used to evaluate pulmonary structure and function. Chest X-rays produce images of the lungs to evaluate symptoms of shortness of breath or chronic cough. Chest X-ray images may show enlarged lungs, air pockets (bullae), or a flattened diaphragm in COPD. During the COVID-19 outbreak, diagnosis of both diseases was largely focused on the use of chest X-ray and CT.

Spirometry is the test to determine lung function and diagnose lung disease. It involves a deep breath and forcefully breathing out (exhaling) into a tube connected to a spirometer. A spirometer measures the amount of air taken in and out of the lung and the time is taken and thus determined how quickly you exhale the air. In Pakistan, the SP100 spirometer is used manufactured by a Chinese company. The post-bronchodilator testing is done to confirm asthma in Pakistan. No patient was found with asthma COPD overlap in this study.

### Inclusion and exclusion criteria

The study includes the monthly and seasonal prevalence of asthma and COPD in the study area while excluding studies on both sex-wise and age-wise prevalence of both asthma and COPD in the district D.I.Khan. Moreover, demographic characteristics of the patients (smoking habits, comorbidities) were also not recorded by the hospital authority and thus were not included in this study.

### Statistical analysis

Asthma data was analyzed by Pearson chi-squared test (*X*-squared = 3627, df = 10, *p* < 0.0001) and COPD data Pearson chi-squared test (*X*-squared = 673, df = 10, *p* < 0.0001). Both diseases showed significance difference by month.

### Indoor and outdoor air pollution in the district D.I.Khan

The US AQI (Air Quality Index) characterizes D.I.Khan with the AQI 157 and air level unhealthy and reported main air pollutant known as fine particulate matter (PM_2.5_) in the district [22]. The PM_2.5_ in the air affects people’s health and reduces visibility.

## Results

### Prevalence of asthma and COPD in 2020

**Fig. 1:**
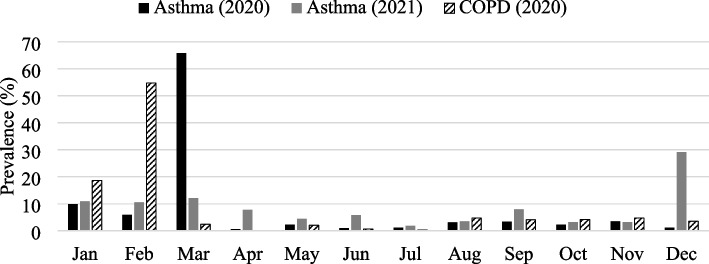
Monthly percentage prevalence of asthma cases in 2020 and 2021 and chronic obstructive pulmonary disease (COPD) cases in 2020 in the District Dera Ismail Khan

March 2020 recorded 1059 (65.8%) cases of asthma (Table 1 and Fig. 1), followed by January 158 (9.8%) cases, and February 94 (5.8%) cases. Asthma has the highest prevalence in early spring (March) followed by mid-winter (January). The remaining each month demonstrated ≤5.8% cases of the total cases. Late spring to early winter showed a relatively low prevalence of asthma. COPD showed the highest prevalence of 405 (54.7%) cases in late winter (February), followed by 138 (18.6%) cases in mid-winter (January) in D.I.Khan.

**Table 1:** Monthly prevalence of asthma (2020 and 2021) and chronic obstructive pulmonary disease (COPD) in 2020 in the District Dera Ismail Khan

Months	No of asthma cases in 2020	No of asthma cases in 2021	Total asthma cases	Percentage prevalence of asthma (total)	No of COPD cases in 2020	Percentage prevalence of COPD
January	158	62	220	10.1	138	18.6
February	94	60	154	7.1	405	54.7
March	1059	69	1128	51.8	18	2.4
April	10	44	54	2.5	0	0.0
May	37	25	62	2.8	15	2.0
June	15	33	48	2.2	5	0.7
July	19	10	29	1.3	2	0.3
August	50	20	70	3.2	35	4.7
September	55	45	100	4.6	30	4.1
October	37	18	55	2.5	31	4.2
November	56	18	74	3.4	35	4.7
December	19	166	185	8.5	26	3.5
Total	1609	570	2179	-	740	-

### Prevalence of asthma in 2021

Table 1 and Fig. 1 indicated asthma’s highest prevalence in December 166 (29.1%) cases and were relatively higher from January through March (winter and early spring) compared to from April through November (late spring to late autumn) in D.I. Khan.

### Prevalence of asthma based on combined data (2020–2021) and yearly comparison

March (early spring) accounted for 51.8% (Table 1) based on the combined data on asthma in D.I. Khan for 2020 and 2021, followed by January (mid-winter: 10.1%) and December (8.5%) demonstrated that the peak season of asthma is the early spring and winter and the lowest in July (mid-summer: 1.3%) in the study area.

Both 2020 and 2021 showed a relatively higher prevalence of asthma in winter and early spring (Table 1 and Fig. 1). Nevertheless, 2020 showed March, while 2021 indicated December as the peak month of asthma prevalence and the lowest prevalence of asthma in April (2020) and July (2021) as both months demonstrated 10 cases each.

### Comparative percentage prevalence of asthma and COPD

**Fig. 2:**
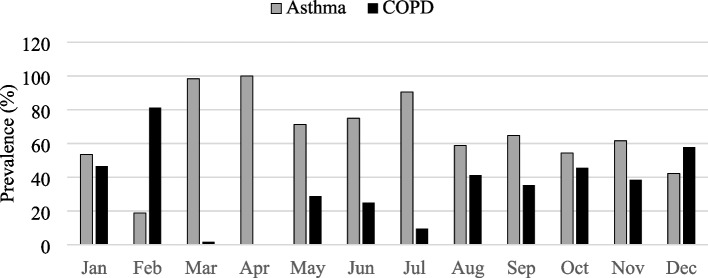
Monthly comparative percentage prevalence of asthma and chronic obstructive pulmonary disease (COPD) in the District Dera Ismail Khan in 2020

The monthly comparative percentage prevalence of asthma and COPD in 2020 (Fig. 2) indicated asthma has a higher prevalence in January and from March through November than COPD in the study period.

## Discussion

Asthma and chronic obstructive pulmonary disease (COPD) both showed a reduced rate of air in and out of the lung because of inflammation in the bronchus/bronchioles and alveoli in the lung [23, 24]. Nevertheless, asthma is characterized by airway obstruction that is typically fully reversible, while COPD is not fully reversible [24]. Determination of the monthly and seasonal prevalence of disease help in understanding its dynamics in populations and the basis for researching other etiological factors to adopt/develop preventive strategies for control of the disease [10, 25].

The present study is unique because it reported for the first time monthly/seasonal prevalence of asthma in KP as well as COPD in Pakistan. Thus, the study added to the existing knowledge of the epidemiological condition of asthma in KP and COPD in Pakistan. Moreover, the study is helpful to clinical practice as it can be used to understand the pathogenesis of both diseases, improve diagnostic accuracy, and help the patient to reduce risk factors, and the physician chooses the correct therapeutic approach.

Updated literature on the epidemiology of respiratory diseases in Pakistan is not available [26, 27]. Although sufficient literature is available on the sex and age-wise prevalence as well as on historical (yearly) trends in the prevalence of asthma and COPD and their risk factors. Nevertheless, little information is available on the monthly and seasonal prevalence of asthma and COPD globally and particularly in Pakistan. Literature on the monthly and seasonal prevalence of asthma and COPD is not available in KP and Pakistan, respectively. Moreover, literature on the comparative percentage prevalence of asthma and COPD is not available globally.

The present study showed the highest prevalence of asthma in March (early spring) and a relatively higher prevalence of both asthma and COPD in winter compared to in the summer or autumn was supported by Raza et al. [21] obtained data from admitted patients of lower respiratory tract diseases in three hospitals in Karachi and found out of total 3205 patients, 53.8% had asthma, 26.4% had COPD, and 19.9% had pneumonia and found clear seasonal pattern showed the highest number of patients admitted in the month of winter (Dec to Feb) and a peak occurring in the spring season (March) for the said diseases. Similarly, Ahmed et al. [28] also collected data from hospitals in Karachi (Pakistan) during 2011–2012 and recorded a total of 2503 patients with asthma admitted which showed seasonal episodes of asthma increased from mid of December to February (winter season), with a peak occurring in March (early spring) and significantly fewer cases of asthma exacerbations occurred in May (summer) and November (autumn). Moreover, Ahmed et al. [28] also found females showed the highest prevalence (65%) of asthma, as well as patients >55 years of age, showed a 64.8% prevalence of overall asthma.

Other studies described sex and age-wise prevalence as Khan et al. [29] found an overall higher prevalence of asthma among boys and children aged 3–7 years in Karachi during 2012–2013. Sultana et al. [27] found pulmonary tuberculosis (29.66%) followed by asthma (28.08%) and COPD (11.31) in Faisalabad during 2013–2014. Khan et al. [5] investigated 105 patients with allergic asthma including males 62 (59.05%) and females 43 (40.95%) in Rawalpindi during 2014–2016. The overall mean age for males and females was 29.9±10.2 years and 28.7±7.0 years, respectively. Mustafa et al. [30] reported an overall 3180 children responded to the questionnaire including males 1767 (56%) and females 1413 (44%), and 71% of them were aged 4 to 11 years. Nocturnal asthma was found in 177 children including 99 (56%) boys and 78 (44%) girls. Batool et al. [31] recorded 116 asthmatic patients including 48% males and 52% females, and 72% of the patients were of age group (60–65 years).

Noman et al. [10] conducted research in the Department of Community Medicine, Gomal Medical College, D. I. Khan, Pakistan, in 2015 and reported 200 asthmatic patients including males 123 (61%) and females 67 (39%). The highest prevalence of 94 (47%) cases was recorded in the age group of 20–29 years, followed by 40 ( 20%) cases in the age group (30–39 years), 23 (11%) cases in the age group (50–59 years), 60–69 years were 11 (6%) cases, and 70–80 years were 12 (6%) asthmatic cases. Majeed et al. [32] reported children who suffered from asthma were aged between 12 months and 8 years and 60% were males who suffered from the disease.

COVID-19 (coronavirus disease 2019) negatively affected the diagnosis services of respiratory diseases globally due to the potential risk of disease transmission during lung diagnosis tests.

## Conclusions

Overall asthma was recorded 73.8% in 2020 and 26.2% in 2021, and COPD was reported 26.2% in 2020. Overall asthma showed the highest prevalence in March (51.8%) and was lowest in July (1.3%). COPD demonstrated the highest prevalence (54.7%) in February asthma relatively more prevailed during winter and early spring and COPD in mid and late winter. Asthma revealed a relatively higher prevalence than COPD most of the year.

## Data Availability

All data generated or analyzed during this study are included in the tables of this manuscript.

## Abbreviations

COPD: Chronic obstructive pulmonary disease.
GAR: Global Asthma Report
PHC: Primary health-care
KP: Khyber Pakhtunkhwa
DHQ: District Head Quarter
D.I. Khan: Dera Ismail Khan
CT: Computed tomography
MRI: Magnetic resonance imaging
COVID-19: Coronavirus disease 2019
AQI: Air Quality Index
PM: Particulate matter
